# Six weeks of N-acetylcysteine antioxidant in drinking water decreases pathological fiber branching in MDX mouse dystrophic fast-twitch skeletal muscle

**DOI:** 10.3389/fphys.2023.1109587

**Published:** 2023-02-14

**Authors:** Asma Redwan, Leonit Kiriaev, Sindy Kueh, John W. Morley, Peter Houweling, Ben D. Perry, Stewart I. Head

**Affiliations:** ^1^ School of Medicine, Western Sydney University, Sydney, NSW, Australia; ^2^ Murdoch Children’s Research Institute, Melbourne, VIC, Australia; ^3^ School of Science, Western Sydney University, Sydney, NSW, Australia

**Keywords:** mdx, skeletal muscle contraction, NAC, oxidative stress, fiber branching, muscle damage, dystrophy, split fiber

## Abstract

**Introduction:** It has been proposed that an increased susceptivity to oxidative stress caused by the absence of the protein dystrophin from the inner surface of the sarcolemma is a trigger of skeletal muscle necrosis in the destructive dystrophin deficient muscular dystrophies. Here we use the mdx mouse model of human Duchenne Muscular Dystrophy to test the hypothesis that adding the antioxidant NAC at 2% to drinking water for six weeks will treat the inflammatory phase of the dystrophic process and reduce pathological muscle fiber branching and splitting resulting in a reduction of mass in mdx fast-twitch EDL muscles.

**Methods:** Animal weight and water intake was recorded during the six weeks when 2% NAC was added to the drinking water. Post NAC treatment animals were euthanised and the EDL muscles dissected out and placed in an organ bath where the muscle was attached to a force transducer to measure contractile properties and susceptibility to force loss from eccentric contractions. After the contractile measurements had been made the EDL muscle was blotted and weighed. In order to assess the degree of pathological fiber branching mdx EDL muscles were treated with collagenase to release single fibers. For counting and morphological analysis single EDL mdx skeletal muscle fibers were viewed under high magnification on an inverted microscope.

**Results:** During the six-week treatment phase NAC reduced body weight gain in three- to nine-week-old mdx and littermate control mice without effecting fluid intake. NAC treatment also significantly reduced the mdx EDL muscle mass and abnormal fiber branching and splitting.

**Discussion:** We propose chronic NAC treatment reduces the inflammatory response and degenerative cycles in the mdx dystrophic EDL muscles resulting in a reduction in the number of complexed branched fibers reported to be responsible for the dystrophic EDL muscle hypertrophy.

## Introduction

It has been proposed that oxidative stress plays a role in the pathophysiology of Duchenne Muscular Dystrophy (DMD), despite this there is currently no effective antioxidant treatment for DMD (Mosca et al., 2021; Lian et al., 2022). N-acetylcysteine (NAC) is an antioxidant that is approved for use in humans, making it attractive as a potential therapeutic treatment for inflammatory conditions in humans (Mokhtari et al., 2017). In skeletal muscle NAC acts as a scavenger of free radicals and contributes to increasing levels of the powerful endogenous intracellular antioxidant glutathione (Kerksick and Willoughby, 2005). Oral dosing with NAC counteracts oxidative stress in skeletal muscles of humans and mice (Medved et al., 2004; Matuszczak et al., 2005; Terrill et al., 2012; Michelucci et al., 2017) and has been shown to ameliorate respiratory muscle dysfunction in animal models of hypoxic disease (O'Halloran and Lewis, 2017).

Several studies have used the mdx dystrophin deficient mouse, the most used animal model of the destructive human dystrophin deficient muscular dystrophy DMD, to study the chronic effects of oral administration of NAC in drinking water or by intraperitoneal injection on skeletal muscle pathology (Terrill et al., 2012; Pinto et al., 2013; Pinniger et al., 2017). These mdx mouse studies show NAC lowers markers of inflammation and oxidative stress in diaphragm and limb muscles and reduces necrotic damage.

There have been reports of a reduction in body weight gain in mice chronically dosed with NAC in their drinking water (Flurkey et al., 2010; Kim et al., 2013; Pinniger et al., 2017). However, rather than this being an adverse effect of NAC dosing it is commonly accepted that oral NAC supplementation reduces body mass gain by decreasing fat levels (Kim et al., 2013; Cao and Picklo, 2014; Ma et al., 2016) and this effect drops off with continued treatment with NAC (Cao and Picklo, 2014).

Previous studies from our laboratory and others have demonstrated a characteristic feature of the dystrophic pathology in mdx mice and DMD boys are the presence of abnormally regenerated branched limb skeletal muscle fibers which increase in number and complexity with age (Bell and Conen, 1968; Schmalbruch, 1976; Head et al., 1992; Chan and Head, 2011; Massopust et al., 2020; Kiriaev et al., 2021b). We and others have demonstrated the increase of these branched fibers mechanically weakens the muscle in the later stages of the dystrophic disease (Head et al., 1992; Chan and Head, 2011; Head, 2012; Pichavant et al., 2016; Kiriaev et al., 2018).

Since the first reports of the mdx mouse it has been noted that the fast-twitch muscles are more susceptible to eccentric (lengthening) contraction (EC) damage (Head et al., 1992; Moens et al., 1993). This susceptibility to EC damage increases as the dystrophic animal ages (Chan et al., 2007; Chan and Head, 2011; Head, 2012; Kiriaev et al., 2018; Kiriaev et al., 2021a). The reason for this increased susceptibility to EC damage is not clear and there have been several hypotheses put forward to explain this effect (Allen et al., 2016); 1) The absence of dystrophin mechanically weakens the sarcolemma making it more susceptible to rupture during EC, 2) Absence of dystrophin is responsible for cycles of necrosis/regeneration resulting in the increase of abnormally regenerated branched fibers structurally compromising the dystrophic membranes ability to resist EC damage 3) The absence of dystrophin results in an increased susceptibility to oxidative damage triggered by EC or 4) The absence of dystrophin sensitizes the ionic mechanisms of the electrical pathways that trigger muscle contraction to damage from EC. It is likely that the increased susceptibility of dystrophic fast-twitch muscle to EC damage involves some combination of these factors.

To test the efficacy of NAC as a possible treatment for DMD we gave it orally for 6 weeks to growing mdx and control mice and measured its effect on body weight gain, EDL muscle weight, fiber branching morphology, contractile function and response to EC. Its main efficacy was in reducing the number of abnormally branched fibers and reversing the increased muscle mass which results from the proliferation of branched fibers as the dystrophic phenotype progresses.

## Materials and methods

### Ethics approval

Animal use was approved by the Western Sydney University Animal Care and Ethics Committee (A14350). Experiments were conducted in compliance with the animal ethics checklist and ethical principles under which the journal operates.

## Animals

Many previous studies on mdx mice have used a separate in-bred colony of wild-type (WT) mice to act as controls. These WT colonies have been separately in-bred since the discovery of the mdx mouse over 25 years ago. This raises the possibility of novel mutations in the WT control group confounding the interpretation of the data from the mdx dystrophic mouse. In our study littermates are bred to act as control animals for mdx mice. These are the gold standard controls for mdx dystrophic studies as both dystrophin negative and dystrophin positive animals are on identical genetic backgrounds, with the only difference being the mutation in the dystrophin gene on the X chromosome at locus Xp21. Mice were obtained from the Western Sydney University animal facility. Male mice were used in the present study to reflect the sex linked DMD condition in boys. The colony of dystrophic mice and littermate controls (LC) used in this study were second generation offspring of C57BL/10ScSn DMD (mdx) mice. The LC were distinguished from dystrophic mice by genotyping. NAC treatment began at 3 weeks of age, and all mice were sampled at 9 weeks of age. The age of mice and duration of treatment were chosen as they reflect a period when the mdx mice have undergone at least one cycle of necrosis and regeneration. The three-to-nine-week age range in mice can be compared to the growth phase of adolescent in humans (Grounds et al., 2008). Mice were housed individually (1 animal per cage) in an environmentally controlled room with a 12 h light/dark cycle at 20°C–25°C and had access to food and water *ad libitum*.

### NAC treatment

NAC (Sigma-Aldrich, Australia) treatment in drinking water was commenced post weaning at 3 weeks of age. NAC was dissolved in RO water and administered as 2% NAC in drinking water for 6 weeks. To overcome any NAC taste avoidance issues drinking water was flavored with banana and caramel (Australian Food Ingredient Suppliers, AFIS); diluted according to manufacturer’s recommendation (0.1% v/v); and sweetened using 0.1% sucralose (Splenda^®^). Untreated animals received same flavored drinking water without NAC. The four experimental groups (*n* = 6 mice per group) were: 1) mdx NAC treated; 2) mdx untreated; 3) LC NAC treated; and 4) LC untreated. Each mouse was housed individually in its own cage. The cage water dispenser was weighed to determine each individual animals water consumption. The mice were weighed every 3 days.

### Muscle preparation

After the 6 weeks NAC treatment, a mouse was placed in a transparent induction chamber and overdosed with isoflurane delivered at 4% in oxygen from a precision vaporizer. The mouse was removed from the chamber and a cervical dislocation carried out. The left and right fast-twitch EDL muscles were dissected from the hind limbs. Once isolated, the EDL muscle was suspended in an organ bath filled with 100% O2 bubbled Tyrode and tied by its tendons from one end to a dual force transducer/linear tissue puller (300 Muscle Lever; Aurora Scientific Instruments, Canada) and secured to a base at the other end using 6–0 silk sutures (Pearsalls Ltd., UK). The EDL data from each animal was averaged to give *n* = 6 (number of mice used). At all times during the dissection and prior to use the hind limb and muscles were kept under 100% oxygenated Tyrode solution to minimize any post-mortem deficits in initial maximum force (Po). Composition of Tyrode solution in mM (also used as dissection solution): 4 KCl, 135 NaCl, 0.33 NaH2PO4, 1 MgCl2, 10 HEPES, 2.5 CaCl2 and 11 glucose, 0.1% fetal calf serum (FCS).

### Muscle force recordings

The muscle was stimulated to contract isometrically by applying a supramaximal voltage across parallel platinum electrodes (701C stimulator; Aurora Scientific Instruments) running the full length of the EDL muscle. Isometric force recordings and eccentric (lengthening) contractions (EC) were made on a 300C Muscle Lever (Aurora Scientific Instruments, Canada). Force responses were analyzed using the 615 A Dynamic Muscle Control and Analysis software (Aurora Scientific Instruments). At the start of each experiment, the muscle was set to optimal length (Lo) which produces maximal twitch force and maintained at this length throughput the experiment. All procedures were performed at a room temperature (20°C–22°C).

### Initial maximum force (Po) and P_max_

At the start of the contractile experiments a supramaximal stimulus was given at 125 Hz (1 ms pulses) for 1 s and the maximum force produced during the tetanic plateau was recorded as Po, the maximum force output of the muscle at optimal length Lo. For the force frequency P_max_ was obtained from the curve fitted to the sigmoidal equation given below. For the EC studies P_max_ was recorded from the isometric plateau of the first EC.

### Force frequency curve

Force-frequency curves were generated at frequencies 2, 15, 25, 37.5, 50, 75, 100, 125 and 150 Hz. A 30s rest was given between each frequency to minimize the effects of fatigue. A sigmoid curve relating muscle force (P) to stimulation frequency (f) was fitted by using a sigmoidal equation. The curve had the equation.

From the fitted parameters of the curve, the following contractile properties were obtained: Half-frequency (Kf) where the force developed is half the sum of (Pmin) and (Pmax). The Hill coefficient (h) which quantifies the slope of the muscle force frequency sigmoidal curve. These were used for population statistics.

### Eccentric contractions and recovery

A series of eccentric (lengthening) contractions (EC) were then performed on each EDL where the contracted muscle was stretched 20% from Lo. At t = 0s, the muscle was stimulated *via* supramaximal pulses of 1 ms duration and 125 Hz frequency. At t = 0.9s, after maximal isometric force was attained, each muscle was stretched 20% longer than their optimal length at a velocity of 2.4 mm/s then held at this length for 2s before returning to Lo. Electrical stimulus was stopped at t = 5s. The EC procedure was repeated 6 times with 3-min rest intervals in between each EC. On completion of the EC protocol, recovery force was measured *via* an isometric contraction given for 1s at 125 Hz (1 ms pulses) at the following time points; 0, 20, 40 and 60 min.

### Muscle mass

After the contractile procedures were completed, the muscle was removed from the transducer, blotted on filter paper, and weighed.

### Skeletal muscle single fiber enzymatic isolation and morphology

EDL muscles were digested in Tyrode solution (without FCS) containing 3 mg/mL collagenase type IVA (Sigma Aldrich,United States), gently bubbled with oxygen, and maintained at 37°C. After 25 min the muscle was removed from solution, rinsed in Tyrode solution containing 0.1% FCS and placed in a relaxing solution with the following composition (mM): 117 KCl, 36 NaCl, 1 MgSO4, 60 HEPES, 8 ATP, 50 EGTA). Each muscle was then gently agitated using pipette suction, releasing individual fibers from the muscle mass. Using a pipette 0.5 mL of solution was drawn and placed on a glass slide for counting. A total of 2025 fibers from 12 EDL muscles were counted: mdx untreated (*n* = 1186 fibers from 6 EDL) vs. mdx NAC treated (*n* = 839 fibers from 6 EDL). Only intact fibers with no evidence of digestion damage were selected for counting. All muscle fibers from LCs showed no branching and were not counted (Kiriaev et al., 2021a).

### Statistical analyses

Data are presented as means ± SD. Differences occurring between genotypes and treatment groups were assessed by two-way ANOVA. Post hoc analysis was performed using Sidak’s multiple comparisons test. Where indicated an unpaired *t*-test was used and significance was accepted at *p* < 0.05. All statistical tests and curve fitting were performed using a statistical software package Prism Version 10 (GraphPad, CA,United States).

## Results

### NAC treatment and water intake, body mass and EDL muscle mass

**FIGURE 1:**
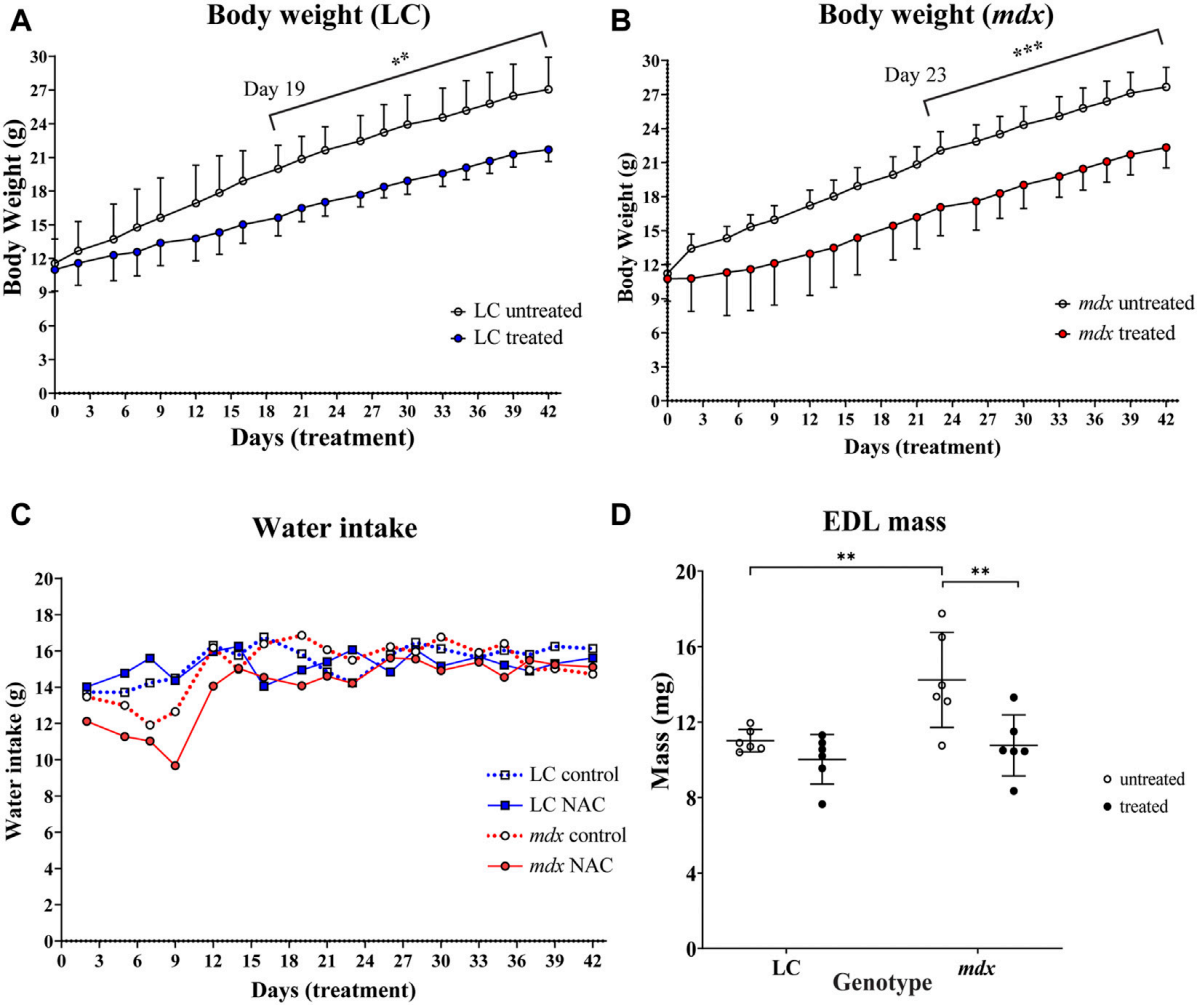
Effect of NAC on body weight **(A)** LC **(B)** mdx **(C)** water intake of animals throughout treatment period and **(D)** muscle mass **(A)** shows significant differences in weight gain from day 19 onward for LCs untreated vs. NAC treated (*p* = 0.0015). **(B)** shows significant differences in weight gain from day 23 onward for mdx mice untreated vs. NAC treated (*p* = 0.0008). **(C)** Line graph showing water intake of all animals throughout the treatment period (error bars omitted for clarity) (*n* = 6) LC untreated/treated and *n* = 6 mdx untreated/treated) **(D)** Scatterplots of EDL muscle mass for LC and mdx untreated and treated groups (n = 6 EDL from each group). Dystrophic EDL muscles from mice not treated with NAC were heavier compared to the treated mdx group (*p* = 0.0034).). Genotype differences between untreated mice show increased muscle mass in mdx EDL compared to LCs (*p* = 0.0062). Data set represent mean value ±SD. Statistical differences displayed within graphs are differences between genotypes and treatment groups assessed by two-way ANOVA, *post hoc* analysis using Sidak’s multiple comparisons test with significance established at *p* < 0.05.

Figure 1 shows the effect on body weight gain, fluid intake and EDL muscle mass of 6 weeks 2% NAC in drinking water. The animals were given the NAC over 3–9 weeks of age which is the period of active growth for mice. Figures 1A, B shows NAC significantly reduced the rate of growth in LC from days 19–42 of treatment (*p* = 0.0015) and mdx animals from days 23–42 of treatment (*p* = 0.0008). Figure 1C shows that the application of NAC to the flavored drinking water did not markedly affect mean fluid intake during the period of the study (for clarity the SD bars have been omitted). Figure 1D shows in LC NAC did not affect the EDL mass, but in mdx NAC treated EDL muscles were significantly lower (*p* = 0.0034) in weight compared to untreated ones. Dystrophic EDL mass was heavier compared to LC (*p* = 0.0062).

### Force-frequency curves and specific Po

**FIGURE 2:**
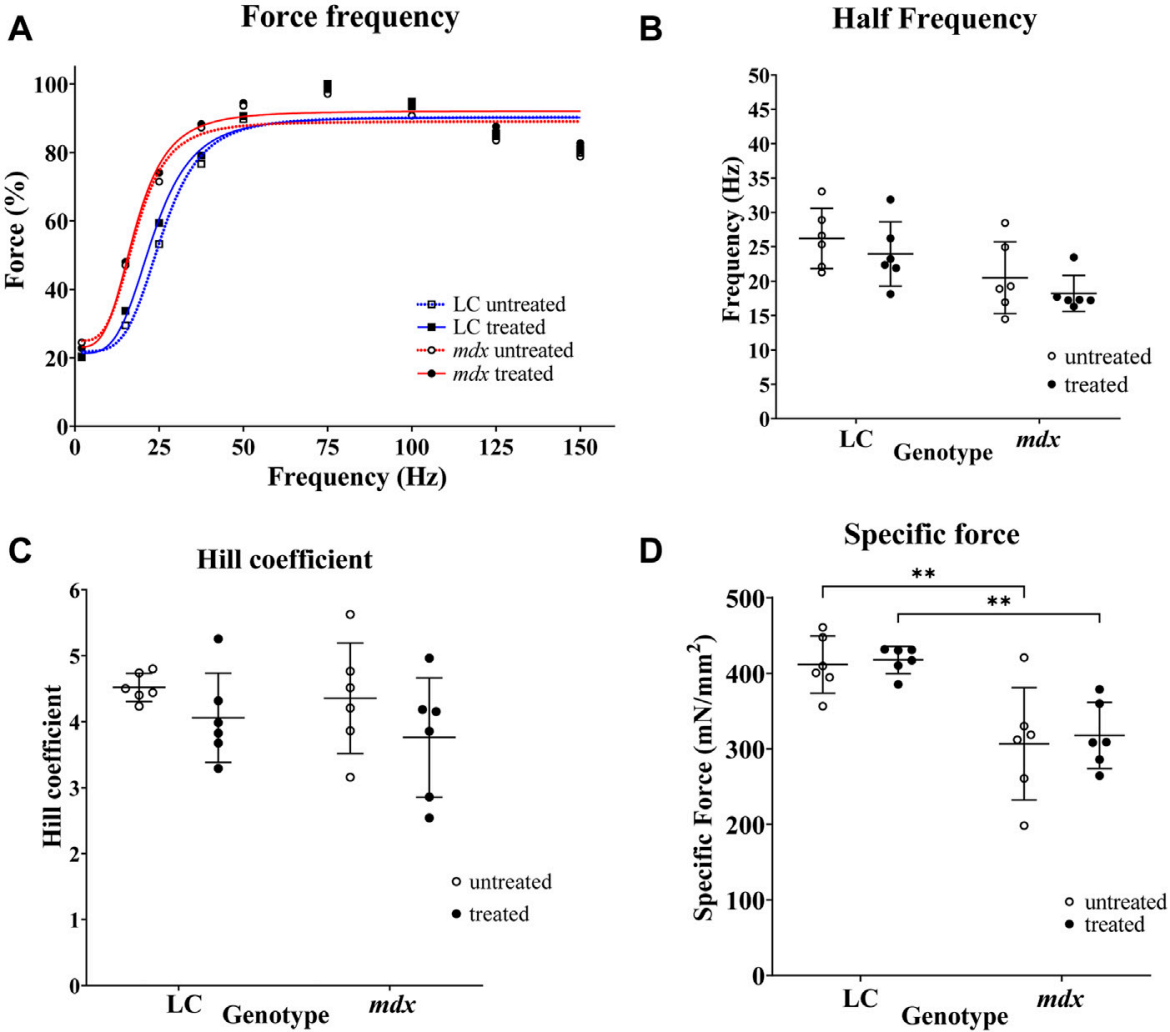
Force frequency curves and fitted parameters and specific force. **(A)** Force frequency data from individual EDL muscles were aggregated to produce a single curve for LC and mdx mice to visualize differences between treatment groups and genotypes (*n* = 6 EDL for each group). Forces are normalized to P_max_. In graph **(A)** SD error bars are omitted for clarity. **(B,C)** are scatterplots of half-frequency and Hill coefficient obtained from force frequency curve shown in **(A)**. **(D)** Shows the maximum specific force (Po) produced by the EDL muscles, there was no effect of NAC treatment, however, as has been reported previously the dystrophin deficient mdx muscles produced significantly less force than LCs, both in NAC treated (*p* = 0.0035) and untreated (0.0023) conditions. Data set represent mean value ±SD.

The aggregate force–frequency curves for NAC treated and non-treated LC and mdx EDL muscles are shown in Figure 2. These curves are fitted with the sigmoidal equation given in the methods, the following best-fit parameters defining these curves were obtained from the group data, half frequency which produced 50% force and the Hill coefficient which is the slope of the rising portion of the sigmoidal curve Figures 2B, C. In Figure 2A forces are expressed as a percentage of the pre-EC P_max_. There were no significant differences in half frequency and Hill coefficient with respect to genotype or treatment (Figures 2B, C). Figure 2D shows the maximum specific force (Po) produced by the EDL muscles, there was no effect of NAC treatment, however, as has been reported previously the dystrophin deficient mdx muscles produced significantly less force than LCs, both in NAC treated (*p* = 0.0035) and untreated (0.0023) conditions.

### Percentage force loss resulting from a series of six ECs at 20% stretch from Lo

**FIGURE 3:**
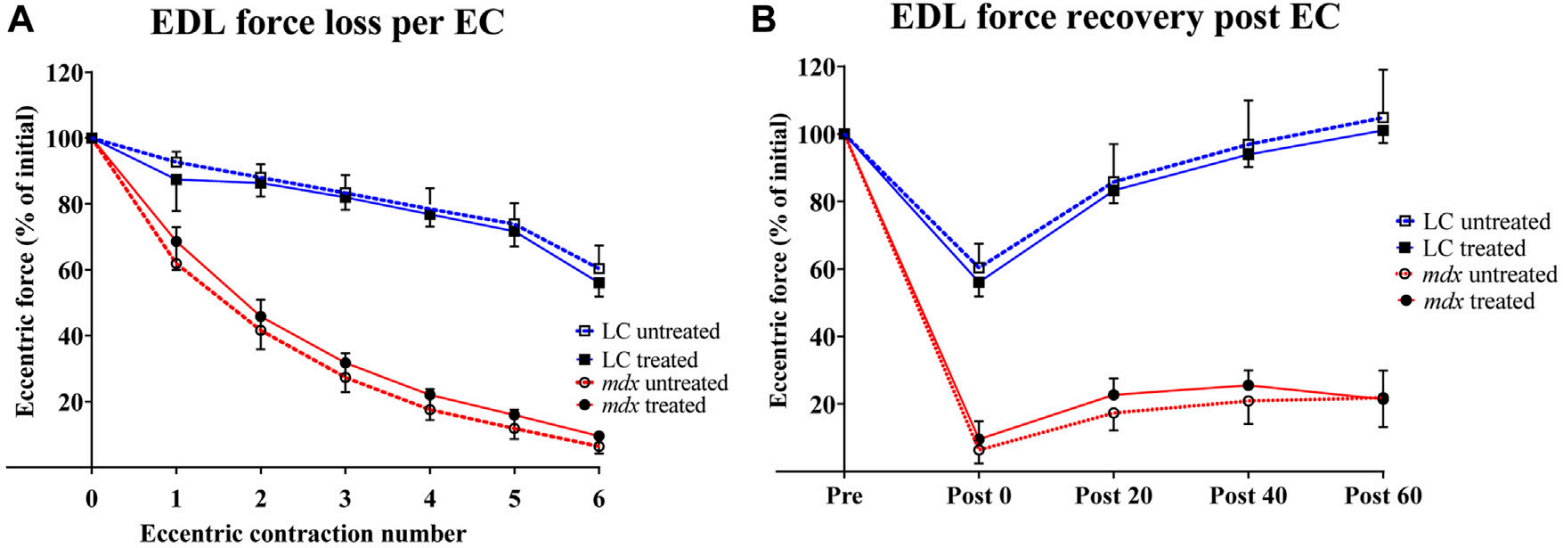
Percentage force loss and force recovery from EC protocol **(A)** Percentage force loss resulting from a series of six ECs at 20% stretch at 2.4 mm/s from Lo (*n* = 6). Force was normalized to the isometric plateau of the first EC in each group **(B)** Percentage force recovery recorded at 0, 20, 40, 60 -minute intervals post EC (*n* = 6). Data shown in both graphs are mean ± SD.

In Figure 3 force was normalized to the isometric plateau of the first EC, prior to stretching to Lo+20% for *n* = 6 EDL muscles of each group. Figure 3A shows that there was a graded reduction of force with each EC for both NAC treated and untreated muscles. As has been previously reported mdx muscle were significantly more susceptible to EC induced force loss when compared to age matched LC (Kiriaev et al., 2018; Kiriaev et al., 2021a) and this was not altered by treatment with NAC. Figure 3B shows the rate of recovery of force over 60 min, mdx muscles recovered ∼20% and this recovery was not different in EDL muscles from NAC treated mdx. In contrast Figure 3B shows EDL muscle from both NAC treated and non-treated LC animals recovered ∼100% force over 60 min.

### Fiber branching morphology in mdx

**FIGURE 4:**
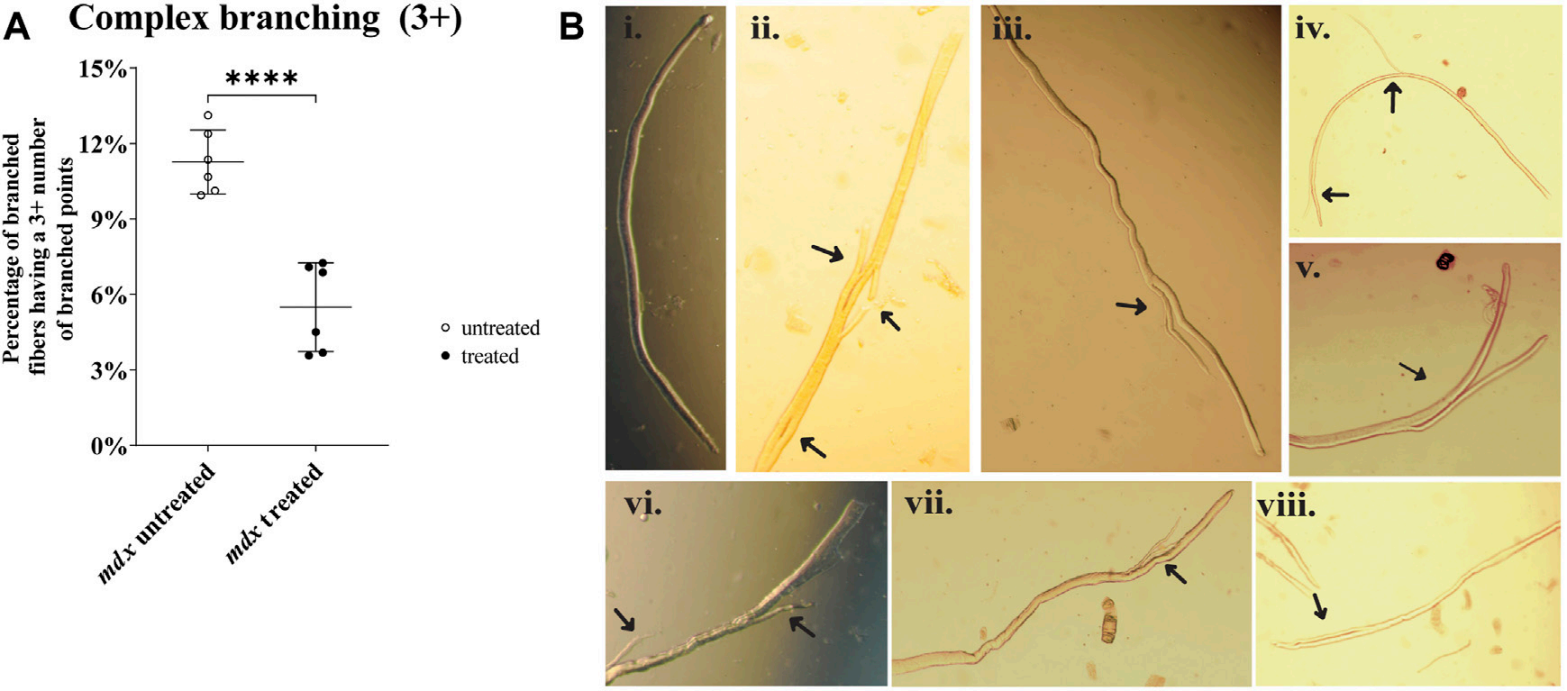
Enzymatically digested mdx EDL muscle fibers. **(A)** Scatterplots of complex fiber branching (3 +) between mdx untreated and mdx NAC treated (P= <0.0001). Fibers from LC were omitted due to no presence of fiber branching. Data shown in both graphs are mean ± SD. Statistical differences displayed in graph are assessed by unpaired *t*-test with significance established at *p* < 0.05 **(B)** Photomicrographs (X200) of enzymatically digested EDL muscle fibers (note in some cases the whole fiber is not shown.) a selection of NAC treated and untreated mdx are shown in **(Bi-viii) (Bi)** a straight mdx fiber with no branching. **(Bii)**: is a fiber with 3 branches **(Biii,v,vii)**: fibers with one branch **(Biv,vi)** fibers with 2 small branches **(Bviii)**: fiber has a split that is connected to the main **(i,iii,iv,v,vii)** are from mdx NAC; **(ii,vi,viii)** are from mdx untreated mice.

Figure 4A compares complex fiber branching (3 + branches) between mdx untreated and mdx NAC treated (P= <0.0001). Figure 4B includes photomicrographs showing an example of a non-branched fiber Figure 4Bi and examples of fiber branching of differing complexity Figures 4Bii–viii.

## Discussion

### The effect of 6 weeks of 2% NAC in drinking water on body weight, muscle mass, and water intake

There have been findings of reduced body mass gain in NAC-supplemented rodents (Kim et al., 2006; Kondratov et al., 2009; Kim et al., 2013; Cao and Picklo, 2014). In particular (Pinniger et al., 2017) using 2% NAC in drinking water for 6 weeks concluded that the reduction of body weight gain in mdx mice was a red flag for considering the use of NAC as a treatment for boys with DMD. Commenting on Pinniger et al., 2017 study (O'Halloran et al., 2018), suggested that some of the reduced weight gain may be attributed to a decrease in body fat and not because of a reduction in lean mass and also that the acidification of the drinking water resulting from the addition of NAC may reduce fluid intake, accounting for some of the weight loss. To address this latter concern, we measured the fluid intake in NAC treated animals compared with no NAC and found no significant effect of NAC on fluid consumption. Confirming Pinniger et al., 2017 finding we also reported that 6 weeks of 2% NAC drinking water caused significant reduction in EDL muscle mass. It is unlikely that this reduction in muscle weight is entirely due to reduced lean body mass, as NAC treatment in LC mice did not cause a significant reduction in the weight of EDL muscles (Figure 1D). As mentioned above a component of the reduction in body weight gain with NAC supplementation has been shown to be due to NAC reducing fat (Kim et al., 2006) and increasing energy expenditure (Ma et al., 2016). It has been shown that fiber branching is responsible for the hypertrophy in the mdx mouse (Faber et al., 2014), so it is reasonable to hypothesize that a proportion of the reduction in EDL muscle mass we show here (Figure 1D) could be a consequence of the reduction in complex 3 + fiber branching we report (Figure 4A).

NAC does not reduce the susceptibility to EC force loss seen in dystrophin deficient fast-twitch muscles, nor does it improve recovery post EC force loss.

The fast twitch EDL muscles of mdx mice have long been known to be more susceptible to damage from EC compared to age match WT controls, as measured by a reduction in Po post EC (Head et al., 1992) and this was confirmed in later studies which utilized LCs for the mdx mice (Kiriaev et al., 2018). In the present study we use a strong eccentric contraction protocol that produced a ∼90% Po force loss in mdx which only recovered to ∼20% of Po after 60 min demonstrating that it was likely the EC had caused significant membrane damage (Olthoff et al., 2018). In contrast, the same EC protocol applied to age matched LC EDL muscle resulted in only a ∼40% force loss which recovered to ∼100% max Po after 60 min, suggesting that no significant membrane damage had occurred in LC as a result of the EC protocol. In the LC the rapidly reversible component of EC force loss fits well with a fatigue induced and/or a reversible ROS-mediated inhibition of contractile force and we conclude there is no muscle damage connected with insults to the sarcolemma integrity. In contrast, Post EC in the mdx muscle there was only 10% Po remaining which recovered to only 20% after 60 min, leading us to surmise that only 10% of the 90% EC induced force loss in the mdx was fatigue induced and/or a reversible ROS-mediated inhibition of contractile force, while the remaining 80% Po deficit is likely muscle damage connected with insults to the sarcolemma integrity disrupting its electrical and ionic control pathways. Six weeks treatment with 2% NAC did not ameliorate the EC induced force loss or improve recovery post EC either in LCs or mdx, in keeping with (Whitehead et al., 2008) who showed 6 weeks of treatment with NAC in drinking water did not protect from eccentric damage at 35°C and there was only a small ∼7% significant improvement at room temperature. We hypothesize that the reduction in complex branching resulting from NAC treatment we report here was not sufficient to reduce the dystrophic muscle below the branched fiber “tipping point” (Chan and Head, 2011; Head, 2012; Kiriaev et al., 2021a). Supplementary Table S2 shows the absolute tetanic force, absolute twitch force, specific twitch force of NAC treated and untreated animals with respect to genotype before EC, after EC and recovery 60 min post EC. The twitch kinetics; time to peak (TTP) and half relaxation time (HRT) for NAC treated and untreated animals with respect to genotype for each of the experimental timepoints are also given in Supplementary Table S2. There was no effect of NAC treatment on any of the parameters.

## Mdx EDL fiber branching in singly housed mice

When compared to our earlier studies (Kiriaev et al., 2018; Kiriaev et al. 2021a; Kiriaev et al. 2021b) the amount of complex 3 + fiber branching we report here from 9-week-old mdx is similar to the mdx EDL muscles from >4 months mdx, see Supplementary Figure S1 for graphical comparison. These current mdx EDL muscles from 9-week-old mice show a force deficit comparable with the mdx EDL muscles from mice aged >4 months. Additionally, 60 min after the EC protocol these current mdx EDL muscles from 9-week-old mice regained a similar amount of force to the mdx EDL muscles from mice aged >4 months. A major difference with our earlier study, where mice were housed ∼ six per cage, is that in this current study mice were housed, one animal per cage. Here this single housing is associated with an accelerate dystrophic branching phenotype. It is possible that single housing may predispose the male mdx mice to greater locomotory activity, possibly associated with the increased space available per animal (Krohn et al., 2006), which would accelerate the appearance of the branching phenotype. It has long been reported that contractile activity is associated with muscle damage in the dystrophinopathies, in fact studies have demonstrated that if you immobilize dystrophic muscles, they do not undergo pathological changes (Allen et al., 2016). While we cannot rule out the possibility that the reduction in branching we see in mdx NAC treatment is due to NAC acting to reduce motor activity by reducing anxiety and stress, it would appear unlikely as a systematic review of clinical trials involving NAC reported that NAC does not reduce anxiety (Deepmala et al., 2015). Additionally, it has been demonstrated that single housing of male mice causes less stress when compared with group-housing of male mice (Kamakura et al., 2016). It is important to note that the force loss and recovery after EC is related to the degree of branching and not the chronological age of the mdx mouse, which supports our long-standing hypothesis that it is the degree of branching which is responsible for the increased EC damage with age (Chan and Head, 2011; Head, 2012; Kiriaev et al., 2018). Supplementary Figure S1 and Supplementary Table S1 uses data from our earlier studies (Kiriaev et al., 2018; Kiriaev et al. 2021a; Kiriaev et al. 2021b) to illustrate the significant increase in 3 + complex branching that occurs from 3 weeks to >112 weeks of age. It is clear that while the degree of motor activity may have a small effect on complex 3 + branching between 9 and 16 weeks, the major phenotype seen across the lifespan of the mdx mouse is the transition from non-branched muscle fibers to the point where >80% of fast-twitch fibers have 3 + complexed branched fibers (Supplementary Figure S1 and Supplementary Table S1), with many fibers containing 10 or more branches (Kiriaev et al., 2021a; Kiriaev et al., 2021b).

Fiber branching, a marker of pathology in mdx muscle, is reduced in fast-twitch EDL muscles from NAC treated mdx.

We and others have shown that a striking pathological feature of regenerated skeletal muscle in the dystrophinopathies is the presence of abnormally branched fibers which dramatically increase in both number and complexity with age (Bell and Conen, 1968; Schmalbruch, 1976; Head et al., 1992; Chan and Head, 2011; Head, 2012; Massopust et al., 2020; Kiriaev et al., 2021a). The number and complexity of these fibers can be used as a marker of the progression of the dystrophinopathies (Bell and Conen, 1968; Kiriaev et al., 2021a). The presence of centrally nucleated (CN) fibers is commonly used as a measure of regeneration, however, it is accepted that by > 8 weeks of age over 90% of mdx mouse skeletal muscle fibers will have regenerated. From 24 weeks to >104 weeks mdx skeletal muscle fibers are 100% centrally nucleated (Massopust et al., 2020), and thus unlike fiber branching, which continues to increase in complexity (Kiriaev et al., 2021a) CN is not reliable indicator of the number of cycles of degeneration/regeneration. Here we show NAC treatment reduces the complexity of branched fibers in the fast-twitch mdx EDL muscle, demonstrating that NAC treatment improves mdx fast-twitch muscle pathology. In the dystrophinopahties it has been proposed that the absence of the protein, dystrophin, from the inner surface of the sarcolemma predispose the skeletal muscle to increased oxidative stress and free radical triggered necrosis, which has been identified as a major cause of muscle injury (Disatnik et al., 2000; Mosca et al., 2021; Lian et al., 2022). The reduction in fiber branching we report here is likely due to NAC reducing (Rando, 2002) the level of free radical triggered necrosis in the active phase of the disease. The reduction in branching we report here was not sufficient to confer a protective effect from the force deficit caused by EC (Chan and Head, 2011). As the mdx mouse model is characterized by a period of florid necrosis 3–20 weeks of age (Duddy et al., 2015) many biochemical changes which are indicative of oxidative damage could be a secondary result of the necrosis, inflammatory response, and subsequent regeneration (Rando, 2002). However, the fact that acute NAC application to the organ bath in *in vitro* experiments has shown that NAC can prevent eccentric contraction force loss in fast twitch mdx muscles (Whitehead et al., 2008; Olthoff et al., 2018; Lindsay et al., 2020) supports the hypothesis that the absence of dystrophin directly increases the susceptibility the muscle to oxidative triggered damage (Mosca et al., 2021; Lian et al., 2022). The present study provides further support for this in finding that 2% NAC in drinking water for 6 weeks prevents the increase in muscle mass characteristic of the dystrophinopathies and reduces the number of complex 3 + fiber branching. These are effects which could be predicted to occur if NAC does indeed reduce myonecrosis (Head, 2012).

## Summary

In the current study we confirmed that 6 weeks of 2% NAC in drinking water significantly reduces body weight gain in both mdx and LC mice. This was not the result of a reduced fluid intake, as was reported in (Kondratov et al., 2009), because we monitored water intake and after some initial fluctuation it was the same for NAC treated mdx and LCs compared to non-treated mdx and LCs. We showed that NAC prevents the increase in skeletal muscle mass characteristic of the dystrophinopathies and reduces the number and complexity of branched fibers which have been shown to be responsible for this increase in mass (Faber et al., 2014; Froehner et al., 2015). This reduction in fiber branching suggests that the antioxidant action of NAC may have reduced the myonecrosis involved in the degeneration/regeneration cycles characteristic of the mdx skeletal muscle pathology. We have previously shown as the number of these cycles increases with age so does the number and complexity of branched fibers in the mdx EDL (Chan and Head, 2011; Kiriaev et al., 2018). However, in our present study NAC does not ameliorate the sensitivity of mdx dystrophic fast-twitch EDL muscle to damage caused by EC (Head et al., 1992) possibly because the reduction in 3 + complex branched fibers is not of sufficient magnitude to be protective.

## Data Availability

The raw data supporting the conclusion of this article will be made available by the authors, without undue reservation.

## Abbreviations

ANOVA, Analysis of Variance; CN, Centrally nucleated; DMD, Duchenne Muscular Dystrophy; EC, Eccentric Contraction; EDL, Extensor Digitorum Longus; f, Stimulation frequency; FCS, Fetal Calf Serum; h, Hill coefficient; HRT, Half relaxation time; Kf, Half frequency; LC, Littermate controls, Lo, Optimal length; NAC, N-acetylcysteine; P, Force developed, Po, Initial Maximal Force from a single 2 s tetani at 125 Hz; Pmax, Maximal tetanic force; Pmin/Pmax, Force developed at minimum/Force developed at maximum; SD, Standard Deviation; TTP, Time to peak.
