# Supplementary material for: Six weeks of N-acetylcysteine antioxidant in drinking water decreases pathological fiber branching in MDX mouse dystrophic fast-twitch skeletal muscle
Source: Front Physiol. 2023 Feb 14;14:1109587. doi: 10.3389/fphys.2023.1109587 (PMC9971923; doi:10.3389/fphys.2023.1109587)
Supplement: Supplementary file 1 [file Table1.pdf]

SUPPLEMENTARY TABLE S1

Kiriaev et al. 2018

Šídák's multiple comparisons test	One-way ANOVA interaction	P-value (multiple comparisons)
2-3 week vs. 6-9 week	****	ns
2-3 week vs. 58-112 week		****
6-9 week vs. 58-112 week		****

Kiriaev et al. 2021

Šídák's multiple comparisons test	One-way ANOVA interaction	P-value (multiple comparisons)
4 month vs. 9 month	****	****
4 month vs. 15 month		****
4 month vs. 18 month		****
4 month vs. 22 month		****
9 month vs. 15 month		*
9 month vs. 18 month		NS
9 month vs. 22 month		**
15 month vs. 18 month		NS
15 month vs. 22 month		NS
18 month vs. 22 month		NS
