# Supplementary material for: Six weeks of N-acetylcysteine antioxidant in drinking water decreases pathological fiber branching in MDX mouse dystrophic fast-twitch skeletal muscle
Source: Front Physiol. 2023 Feb 14;14:1109587. doi: 10.3389/fphys.2023.1109587 (PMC9971923; doi:10.3389/fphys.2023.1109587)
Supplement: Supplementary file 2 [file Table2.pdf]

Supplementary table S2

		LC untreated	LC treated	*mdx* untreated	*mdx* treated
Maximum absolute tetanic force (mN)		329.28 ± 20.91	307.66 ± 31.92	299.79 ± 24.14	256.98 ± 33.83
Twitch force (mN)	Pre EC	50.44 ± 7.42	47.16 ± 6.17	52.89 ± 10.69	42.38 ± 6.06
	Post EC	36.09 ± 4.26	30.94 ± 3.12	6.03 ± 4.86	6.20 ± 2.13
	Recovery	32.52 ± 3.88	27.90 ± 3.47	6.57 ± 4.44	5.24 ± 2.64
Twitch specific force (mN/mm^2)	Pre EC	57.41 ± 9.98	58.46 ± 6.75	49.07 ± 17.06	49.19 ± 9.89
	Post EC	40.99 ± 5.20	38.54 ± 4.96	5.64 ± 4.57	7.29 ± 3.26
	Recovery	36.92 ± 4.75	34.71 ± 4.57	6.03 ± 4.11	6.21 ± 4.17
Time to peak (ms)	Pre EC	34.08 ± 2.23	35. 42 ± 2.63	36.33 ± 4.82	35.71 ± 1.85
	Post EC	29.33 ± 1.64	29.21 ± 1.98	27.29 ± 1.55	27.75 ± 1.05
	Recovery	23.92 ± 1.13	23.71 ± 1.06	22.75 ± 1.10	21.04 ± 3.97
Half relaxation time (ms)	Pre EC	25.67 ± 4.69	30.33 ± 3.13	35.33 ± 9.38	40.04 ± 5.67
	Post EC	22.29 ± 1.94	21.79 ± 1.86	22.29 ± 2.49	22.96 ± 1.22
	Recovery	16.38 ± 0.63	16.29 ± 0.89	17.08 ± 1.10	16.63 ± 2.03

All data are displayed as Mean ± SD (n=6 EDL muscles for each of the groups).

Maximum Absolute Force, Twitch force and Kinetics

Supplementary table S2 shows the absolute tetanic force, twitch force, specific twitch force of NAC treated and untreated animals with respect to genotype before EC, after EC and recovery 60 minutes post EC. Twitch kinetics; time to peak (TTP) and half relaxation time (HRT) are displayed for NAC treated and untreated animals with respect to genotype for each of the experimental timepoints. There was no effect of treatment across all parameters.
