# Supplementary figures and images for: Six weeks of N-acetylcysteine antioxidant in drinking water decreases pathological fiber branching in MDX mouse dystrophic fast-twitch skeletal muscle
Source: Front Physiol. 2023 Feb 14;14:1109587. doi: 10.3389/fphys.2023.1109587 (PMC9971923; doi:10.3389/fphys.2023.1109587)

## Supplementary A

### Complex (3+) branching in *mdx* EDL muscles

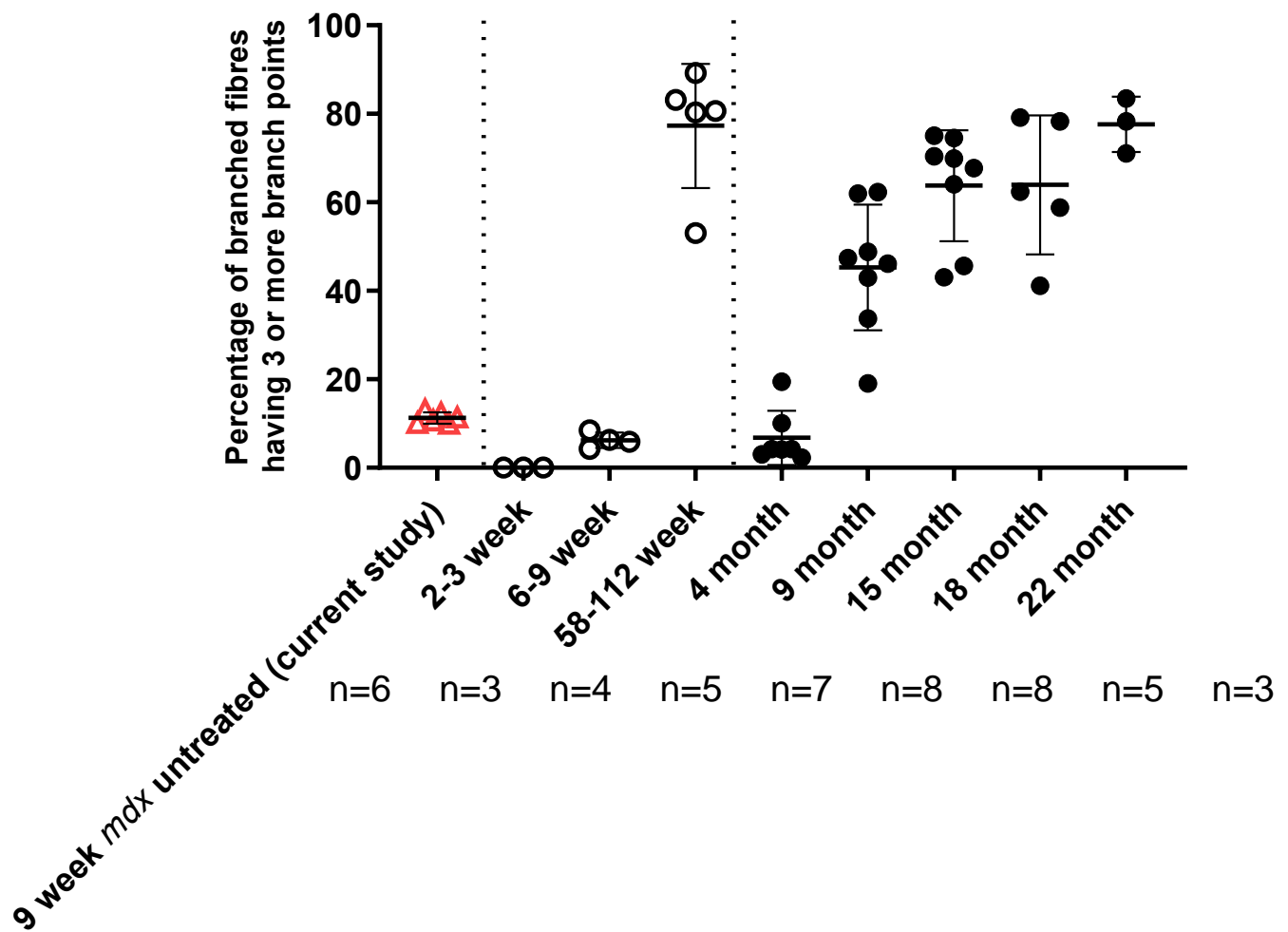

Supplement: Supplementary file 3 [file Image1.pdf]
